# Parametric Study on In Situ Laser Powder Bed Fusion of Mo(Si_1−x_,Al_x_)_2_

**DOI:** 10.3390/ma13214849

**Published:** 2020-10-29

**Authors:** T. Minasyan, S. Aydinyan, E. Toyserkani, I. Hussainova

**Affiliations:** 1 Department of Mechanical and Industrial Engineering, Tallinn University of Technology, Ehitajate 5, 19086 Tallinn, Estonia; sofiya.aydinyan@taltech.ee; 2 Multi-Scale Additive Manufacturing Laboratory, Department of Mechanical and Mechatronics Engineering, University of Waterloo, 200 University Ave., West Waterloo, ON N2L 3G1, Canada; ehsan.toyserkani@uwaterloo.ca

**Keywords:** molybdenum disilicide, aluminum alloy, additive manufacturing, laser powder bed fusion, molybdenum aluminosilicide, parametric study, surface roughness, hardness, density

## Abstract

Mo(Si_1−x_,Al_x_)_2_ composites were produced by a pulsed laser reactive selective laser melting of MoSi_2_ and 30 wt.% AlSi10Mg powder mixture. The parametric study, altering the laser power between 100 and 300 W and scan speed between 400 and 1500 mm·s^−1^, has been conducted to estimate the effect of processing parameters on printed coupon samples’ quality. It was shown that samples prepared at 150–200 W laser power and 400–500 mm·s^−1^ scan speed, as well as 250 W laser power along with 700 mm·s^−1^ scan speed, provide a relatively good surface finish with 6.5 ± 0.5 µm–10.3 ± 0.8 µm roughness at the top of coupons, and 9.3 ± 0.7 µm–13.2 ± 1.1 µm side surface roughness in addition to a remarkable chemical and microstructural homogeneity. An increase in the laser power and a decrease in the scan speed led to an apparent improvement in the densification behavior resulting in printed coupons of up to 99.8% relative density and hardness of ~600 HV1 or ~560 HV5. The printed parts are composed of epitaxially grown columnar dendritic melt pool cores and coarser dendrites beyond the morphological transition zone in overlapped regions. An increase in the scanning speed at a fixed laser power and a decrease in the power at a fixed scan speed prohibited the complete single displacement reaction between MoSi_2_ and aluminum, leading to unreacted MoSi_2_ and Al lean hexagonal Mo(Si_1−x_,Al_x_)_2_ phase.

## 1. Introduction

The research interests for metal silicides have continuously revolved around the development of a composite material with sufficient operating temperatures thresholds employable in applications across the lucrative aerospace and energy production sectors [1]. MoSi_2_ is an attractive transition metal silicide for structural applications at elevated temperatures as it possesses an outstanding oxidation resistance up to 1700 °C [2], increasing with temperature electrical resistivity, which makes the material to be considered as a promising heating element [3]. Moreover, MoSi_2_ exhibits an excellent alloying compatibility when paired with some compounds including metals [4,5], silicides [6], and ceramics [7]. Aluminum is one of the alloying elements negating the pest oxidation of MoSi_2_ through the formation of alumina (or mullite) due to the development of the Mo(Si,Al)_2_ compound [8,9,10]. Manufacturing of Mo(Si,Al)_2_ composites into bulks is a challenging task with the conventional press and/or sintering methods; however, additive manufacturing techniques, which offer flexible fabrication, are prone to overcome many obstacles.

The laser powder bed fusion (LPBF)/selective laser melting (SLM) technique, a class of additive manufacturing (AM), enables the construction of intricately shaped objects. LPBF offers design freedom, while mitigating the problems associated with traditionally produced complex shapes [11]. The technique uses a 3D computer-aided design blueprint constructing 2D cross section slices of the model. As a powder distribution system evenly spreads a thin layer of powder on the build area, a high-power laser selectively melts the powder zones provided by an execution build file, which references consecutive layers of computer-aided design (CAD) model. Each laser cycle produces a new cross section slice of the constructed object; the work platform is incrementally lowered before a new powder layer is spread. The various processing parameters influence the microstructure, mechanical, and even chemical properties of the fabricated parts, which are related to (i) laser (wavelength, power, spot diameter, focal length, transverse electromagnetic mode, beam shape, pulse duration, and pulse repetition rate (if a pulsed laser is used)) [12], (ii) scan (speed, scanning pattern, scan line space, and scanning angle change) [13], (iii) feedstock (particle size, shape and distribution, packing density, layer thickness, powder flowability, melting point, chemical composition, laser absorptivity, etc. [14,15]), (iv) temperature (powder bed or feeder temperature) [15], (v) reaction chamber environment (inert gas type, pressure, flow rate, and direction) [16,17], (vi) build platform (material, melting point, thickness, possible reactivity with powder) [17], (vii) process setup (sample location, build angle, orientation) [18,19], (viii) powder reuse [20,21], and some others.

The effect of various parameters on the quality of LPBF-printed AlSi10Mg and MoSi_2_-based items have recently been widely studied. The influence of scan path and gas flow rate on tensile properties of LPBF-printed AlSi10Mg parts using laser power of 350 W laser, a layer thickness of 100 μm, scanning speed of 900 mm·s^−1^, and hatch distance of 120 µm was reported in [22]. It was shown that scanning against the gas flow nearby a gas outlet and an increase in gas flow rate improve the tensile properties of the material. The optimization of process parameters is given in [23]. The minimal porosity of 0.8% for AlSi10Mg samples was achieved at 175–200 W laser power, 1025–1350 mm·s^−1^ scan speed, and 50–65 µm hatch distance. As aluminum is a highly reflective metal and possesses a high thermal conductivity, a high enough laser power has been recommended for use [16]. 

The AlSi10Mg single tracks were produced by LPBF at a fixed laser power of 180 W and a layer thickness of 35 μm and scanning speed altered in 600 to 1600 mm·s^−1^ range [24]. The fully dense sample was fabricated only at a scanning speed of 1000 mm·s^−1^. In [25], the preparation of MoSi_2_ 10–13 wt.% Si composite lattices applying 36–84 W of laser power and low 80 mm·s^−1^ scan speed was reported. Manufacturing of MoSi_2_ bulks using 100 W laser power and 400–1200 mm·s^−1^ scanning speed showed that the volume fraction of voids enlarged with an increase in the scanning speed hindering the continuous growth of grains along the building direction [26]. The authors’ previous paper [27] reports on a successful reactive powder bed fusion of Mo–Si–AlSi10Mg alloy applying 100 W laser power and 300–500 mm·s^−1^ scanning speed yielding at preparation of almost fully dense Mo(Si_1−x,_Al_x_)_2_ based composite.

In our previous work [28], we reported the in situ preparation of Mo(Si,Al)_2_-based composite bulks by selective laser melting of a MoSi_2_-30 wt.% AlSi10Mg powder mixture covering the powder characteristics, printability, and phase composition of the produced bulks. The main goal of this work is to analyze the impact of process parameters on surface roughness, densification behavior, hardness, and microstructure development of the printed parts to identify the optimum processing window for an efficient materials fabrication. The parametric study considers the laser power and scanning speed as variables of primary importance, which strongly influence the microstructure, surface roughness, and mechanical properties of the samples. Therefore, the current study is mostly concentrated at these parameters leaving other process variables out of scope. The motivation behind the work is the preparation of molybdenum aluminosilicide-based samples with high relative density, low surface roughness, microstructural and compositional homogeneity, as well as sufficient hardness to be used as a structural material or a heating element.

## 2. Experimental

The powder mixture of MoSi_2_ and 30 wt.% AlSi10Mg was used as a feedstock for LPBF process. MoSi_2_ powder was prepared by combustion synthesis of stoichiometric Mo–Si reactant mixture (Mo:Si; 1:2 molar ratio), then mixed with 30 wt.% commercial AlSi10Mg alloy powder by mechanical mixing for 3 h as reported in [28]. Particle size distribution, packing density, and basic flowability energy of the powder are detailed in [28]. Powder cohesion and the flowing angle were measured by GranuDrum (GranuTools, Awans, Belgium). The powder was half filled into the drum rotating around its axis at 2–10 rpm angular velocity. Images were captured after each rotation. The position of air/powder interface or the angle of repose was determined by an edge detection. 

**Figure 1:**
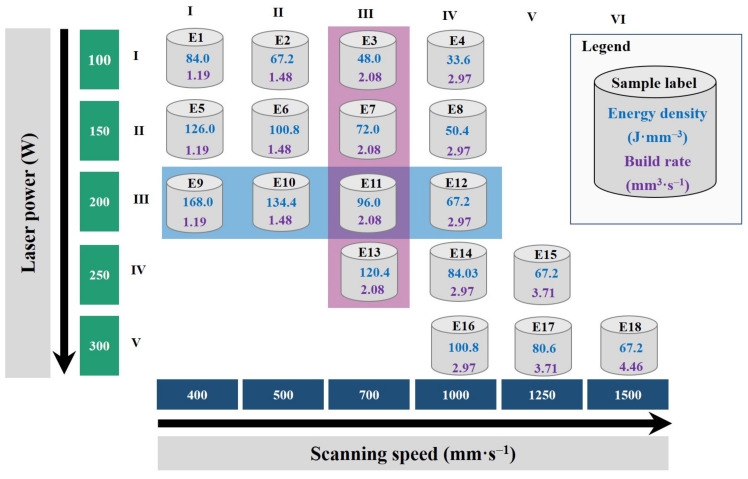
Scheme of used parameters according to scan speed and laser power (Note: The samples highlighted with background blue and purple colors are subjected to microstructural analysis).

A Renishaw AM400 customized machine (Renishaw, Wotton-under-Edge, UK) was used for the consolidation of MoSi_2_-30 wt.% AlSi10Mg mixture. The device possesses an effective build volume (X × Y × Z) of 250 mm × 250 mm × 300 mm and is equipped with a Gaussian distributed ytterbium continuous wave laser with a maximum power of 400 W and wavelength of 1070 nm, modulated to operate as a pulsed laser. Prior to laser initiation, the chamber was filled with argon gas with the oxygen level lower than 500 ppm. A meander scan pattern was used, and the scanning angle was interchanged by 67° upon the antecedent layer. Cylindrical samples with 7 mm × 7 mm were built on a reduced size (100 mm (X) × 100 mm (Y)) aluminum platform. For the parametric study, two parameters, namely, the laser power (*P*) and scan speed (*ν*), were altered. The laser spot size and hatching distance (*h*) were maintained at 85 μm and 90 μm, respectively, while the laser power was changed from 100 W to 300 W with the step size of 50 W. The beam spot diameter at the focused position was 70 µm. To get a beam spot diameter of 90 μm, the focal point of the laser beam was defocused to a point above the build plate of the system. Point distance was chosen 85 μm and exposure time accordingly to achieve 400–1500 mm·s^−1^ scan speed (*ν*). All samples were built using a Z vertical increment of 35 μm (layer thickness (*d*)). The built rate and the laser volumetric energy density (LVED) were derived from *BR = νhd* and *E = P/νhd* equations, respectively. The labels for each sample, the scheme of the corresponding process parameters based on the laser power, scanning speed and the corresponding energy density and build rate are depicted in Figure 1. Three samples were prepared under each condition and subjected for further investigation.

The dimensional accuracy by means of surface roughness was estimated by Keyence VK-X250 laser scanning microscope (Keyence Corporation, Osaka, Japan) using 20× lens. Four measurements were performed for each surface, and the average values of the maximum height (Sz) and the arithmetical mean height (Sa) were taken. 

Two scanning electron microscopes (SEM)—TESCAN VEGA3 (Brno, Czech Republic) and HR-SEM Zeiss Merlin (ZEISS, Oberkochen, Germany) equipped with Bruker QUANTAX 200 and Bruker EDX-XFlash 6/30 (Billerica, MA, USA) EDS detectors, respectively, were used to examine the morphology and chemical composition of the initial powder, as well as the microstructural development in as-built and polished parts.

The phase composition analysis was performed by Rigaku SmartLab SE X-ray diffractometer (Tokyo, Japan) (with CuKα radiation, λ = 1.54056 Å), with 1D detector D/teX Ultra 250. Samples were analyzed at 20–100° 2θ range, 0.01° step size, and 2.5°·min^−1^ measuring speed, using Bragg–Brentano geometry.

Additionally, the splashed powders during laser scanning were studied, as it has a critical role in process outcome. The blown powders located close to the gas outlet were collected after the completion of the whole process and analyzed.

The density of the fabricated specimens was measured by Archimedes’ method (Mettler Toledo ME204, Greifensee, Switzerland) and by calculating samples’ dimensions (by digital Vernier caliper, KS Tools Werkzeuge-Maschinen GmbH, Hessian, Germany, 0.01 mm accuracy) and weight. Relative density was calculated by taking the materials’ bulk density as 4.37g·cm^−3^. 

The hardness was measured at different locations using a Vickers microhardness tester (Indentec 5030 SKV, Stourbridge, UK) with 1 kg and 5 kg loads (9.8 N and 49 N) and 10 s dwell time.

## 3. Results and Discussion

### 3.1. Feedstock Powders

**Figure 2:**
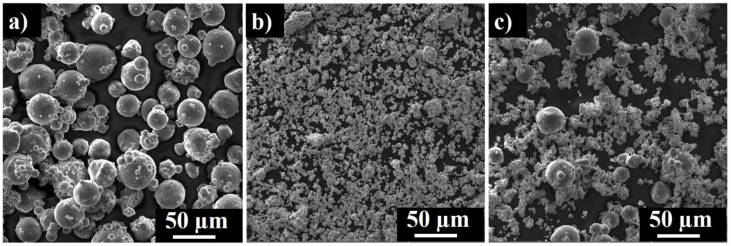
SEM images of AlSi10Mg alloy (**a**), MoSi_2_ (**b**), and MoSi_2_-30 wt.% AlSi10Mg powder mixture (**c**).

Powder feedstock represents 70 wt.% MoSi_2_ and 30 wt.% AlSi10Mg mixture (Figure 2c) with a negatively skewed Gaussian size distribution (D10 = 7.68 µm, D50 = 33 µm, D90 = 54 µm). AlSi10Mg alloy contains spherical and occasional satellite particles with Gaussian size distribution centered on 38 µm (Figure 2a).

The MoSi_2_ powder displays 1–5 micron-sized MoSi_2_ particles with frequent agglomerates (D50 = 18 µm) (Figure 2b).

The angle of repose at 2 and10 rpm drum cell rotation speed was 44.85° and 45.5°, respectively. The cohesion was measured to be 13.25–14.25 highlighting the fair flowability of the powder and applicability for the LPBF process.

### 3.2. Surface Roughness

**Figure 3:**
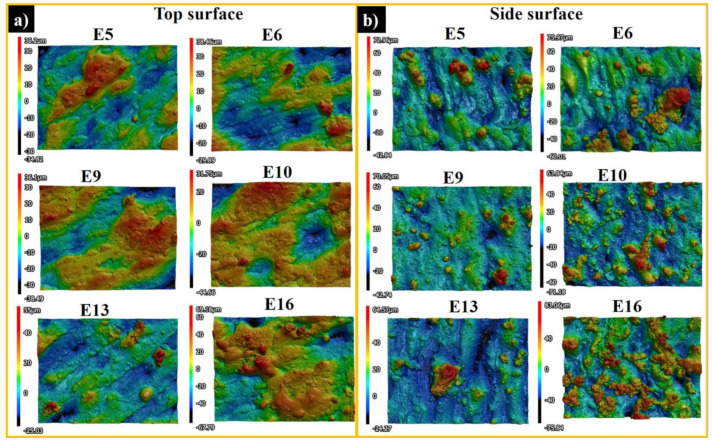
Top (**a**), and side (**b**) surface profiles of samples E5, E5, E9, E10, E13, and E16.

**Figure 4:**
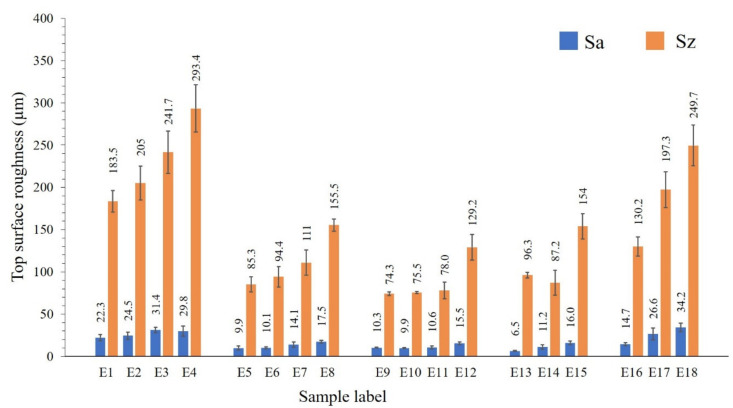
Top surface roughness results of samples E1–E18.

Figure 3 shows the top (a) and side surface profiles (b) of samples E5, E6, E9, E10, E13, and E16. The average top surface roughness (Sa) and the maximum height (Sz) are presented in Figure 4. The *S*_z_ is defined as the sum of the largest peak height and the largest pit depth values within the target areas. The zero level is based on the mean plane. SLM fabrication quality and surface integrity vary significantly depending on different process parameters.

An increase in the scanning speed at a fixed laser power leads to an increase of top surface irregularities and affects the dimensional precision of fabricated parts. This is mainly attributed to the fact that at a higher scanning speed, the likelihood of balling phenomenon, and insufficient fusion could be higher; both resulting in a poor surface quality. However, at the same scanning speed, the samples produced with a higher laser power possess smoother surfaces (e.g., samples E1–E5–E9 and E4–E8–E12) (Figure 4).

The samples produced using the lowest laser power of 100 W (E1–E4) have deep pits and high peaks. This may be conditioned by an insufficient heat for complete melting of consecutive layers causing major “lack of fusion” zones. In this case, the pattern of the scan track is not formed and the portion of the scanned powder layer remains in sintered condition. Moreover, when the powder layer is not completely melted, some of the powder particles become irregular in shape and larger in size, surpassing the set layer thickness and preventing the homogeneous spread of the subsequent virgin powder layer. The thickened layers increase the instability of the pool, weakening the bonding of the pool and the substrate, meantime promoting the balling effect. As a result, as the part fabrication is beyond the melting zone, the relative density and surface quality of LPBF-produced E1–E4 parts are low.

From the top surface profiles of E6 and E13 (Figure 3a), the laser scanning tracks are clearly visible. There are spherically shaped spattered particles at the surfaces of samples E5 and E13, which can potentially be either (i) surface oxidized Al alloy from the initial powder or heat affected zones, (ii) splashed droplets from the melt pool, or (iii) droplets spattered from other printed samples during laser scanning. Because of instability of the melt and an inevitable oxidation phenomenon during the melting process, the melted mass is not sufficiently wetting the underlying layer, which results in a rough surface finish, subsequently obstructing a smooth layer deposition and decreasing the density of the produced part [29]. 

In all probability, the use of higher laser powers and high scan speeds, such as 150–250 W/1000–1250 mm·s^−1^ or 300 W/1000–1500 mm·s^−1^ (Figure 1, samples in columns iv–vi), the balling phenomenon may occur due to capillary and wetting forces between the partially and fully melted particles interfaces.

Relatively smooth top surfaces were built for samples E5, E6, E9, E10, E11, and E14, as Sa was measured to be in the 9 to 11 µm range. The lowest Sa was estimated for the sample E13 (6.5 ± 0.5 µm). When the laser heats the powder layer, there is an obvious temperature difference among the laser beam and the scanned area. This induces shear forces on the molten mass surface, which is counteracted by surface tension forces [30]. After scanning the selected zone, the heat source moves, and the temperature evenly dissipates through the samples. The gravity force and the curved surface then neutralize the external shear force reinstating the leveled surface height of the melt pool. Due to an extended solidification time, this relaxation process will complete with a smooth surface finish [30]. 

A gradual increase is observed for Sz value when the scanning speed is increased, and the laser power is kept constant (Figure 4 and Figure 1 samples in I–V rows). 

Samples E9, E10 and E11, which are produced at 200W laser power and 400–700 mm·s^−1^ scanning speed, demonstrate the low Sa and Sz values. Sample E16 is produced using the highest laser power of 300 W; nonetheless, the surface finish is mediocre as compared to E9–E11 samples. This is associated with (i) the capillary effect and wetting forces changing the thermal domain, as when capillary force is not high enough to grab the particles inside the melt pool, the particles partially melt on the skin, and (ii) fast solidification and an insufficient relaxation time of the melt pool, enduring rippling on the surface and deterioration of surface quality. 

Some other factors, such as leveled powder spreading, unchangeable effective spot diameter, and oxygen level in the chamber, are also decisive to secure a stable manufacturing process.

**Figure 5:**
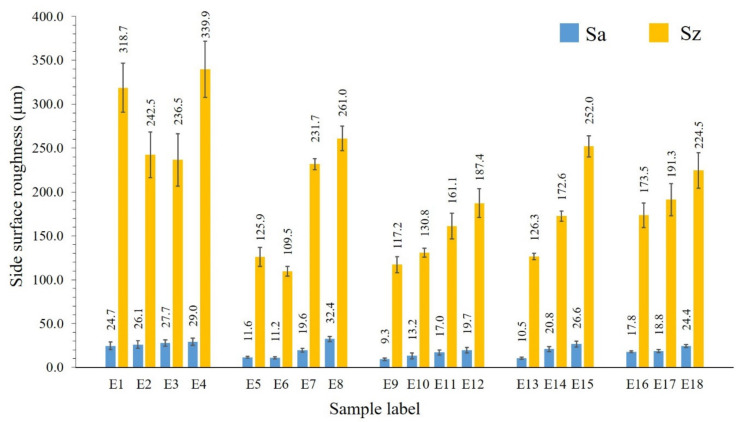
Side surface roughness results for samples E1–E18.

Analogous to the top surfaces, the samples’ side surfaces produced using 100 W laser power show the enlarged roughness (Figure 5). A higher laser power and a lower scanning speed result in a higher quality of side surface, supposing a complete melting/sintering of the outer surfaces. The poor side surface roughness of samples E1–E4 is determined by the incomplete melting of the outer layers. If the outer powder layer is not completely melted/sintered, then the partially melted or unmelted particles may remain attached to the surface. However, in case of a high applied energy density, there are incidences of adhesion of feedstock particles from the partially heated belts occasioning a high surface roughness.

Sa represents the difference of every point’s height in comparison with the arithmetical mean of the surface in the definition area. The low Sa and Sz values are measured for samples E5, E6, E9, E10, and E13 to be in the range of 9.3 to 13.2 µm and 109.5 to 139.8 µm, respectively (Figure 5). Samples printed using 300 W laser power possess comparatively high Sa and Sz values, due to the high number of adhered particles. For the sample E14, a relatively higher laser power was used, which favors the adherence of the powders from the heat affected zones, and relatively high scan speed, decreasing the applied energy per volume. This might cause insufficient fusion of the powder in outer boundary. For E11, as well, the reason of higher side surface roughness can be explained by the attached particles from a powder bed and insufficient heat provided for melting the powder in the outer boundary. As compared to the top surface, the roughness of the side surface is doubled. The adhered particles from the heat affected zone influence not only the maximum height, but also the arithmetical mean height, as demonstrated by this case.

Therefore, the parameters of 150–200 W along with 400–500 mm·s^−1^ scanning speed and 250 W laser power with 700 mm·s^−1^ provide a relatively good surface finish with moderate surface roughness.

**Figure 6:**
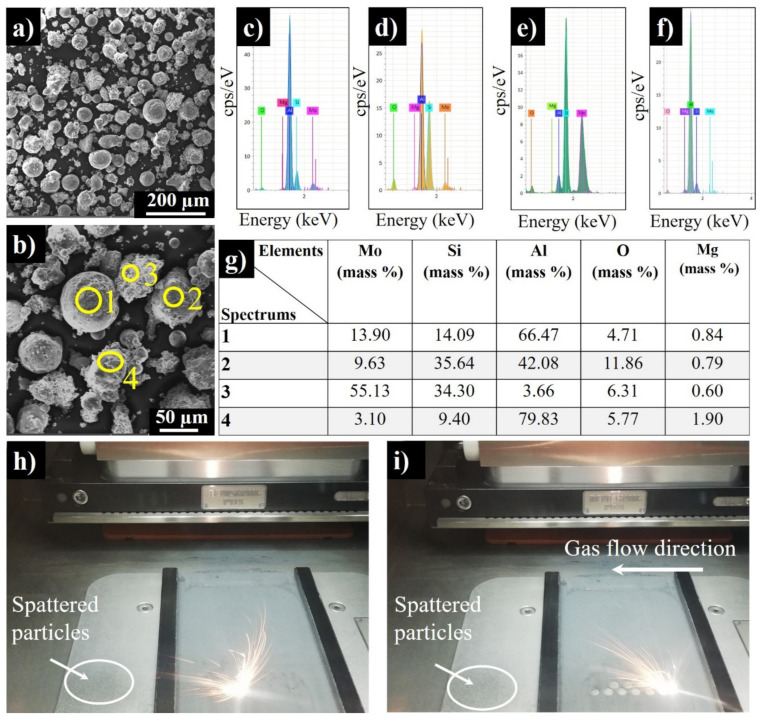
SEM images of spattered powder (**a**,**b**), corresponding EDS spectrums (**c**–**f**), chemical composition of spattered particles 1–4 (**g**), and the spattering observed during the process (**h**,**i**) (Note that the gas flow direction is from the right to the left side of the chamber.).

Additional studies were made to find out the nature of spattered particles, as they were detected in both top and side surfaces of samples (Figure 3a,b). They were collected from the left side of the chamber. Apparently, the right surfaces of the samples are more vulnerable to a higher roughness, as gas flows from right to left blowing away the debris (Figure 6h,i). The SEM images of the spattered powders display that the particles became more acicular in shape and about 1.5–2 times bigger in size (Figure 6a,b) as compared to the virgin powder. The presence of oxygen was detected in the range of 4–11% by EDS. Moreover, Mo, Al, and Si contents have been reduced because of increased oxygen content. The change in powder composition disturbed the balance of Mg amount, as well.

Theoretically, the spherical particles are expected to be originated from the molten AlSi10Mg droplets, and the irregular-shaped ones from MoSi_2_ powder splashing. Spectrums 1–4 (Figure 6c–f) confirm the above-mentioned statement, as the particles of rounded morphology have the high content of aluminum (Figure 6b–f). According to Spectrum 3 (Figure 6e), “Particle 3” contains mostly Mo and Si together with a small amount of Al; however, it contains up to 6% oxygen pointing to the oxidation by residual oxygen in argon. The AlSi10Mg debris has a rough texture with the presence of surface oxides rich with Mg. Mg is also oxidizing due to its instability and has a high affinity towards oxygen [31].

### 3.3. Microstructural Analysis

**Figure 7:**
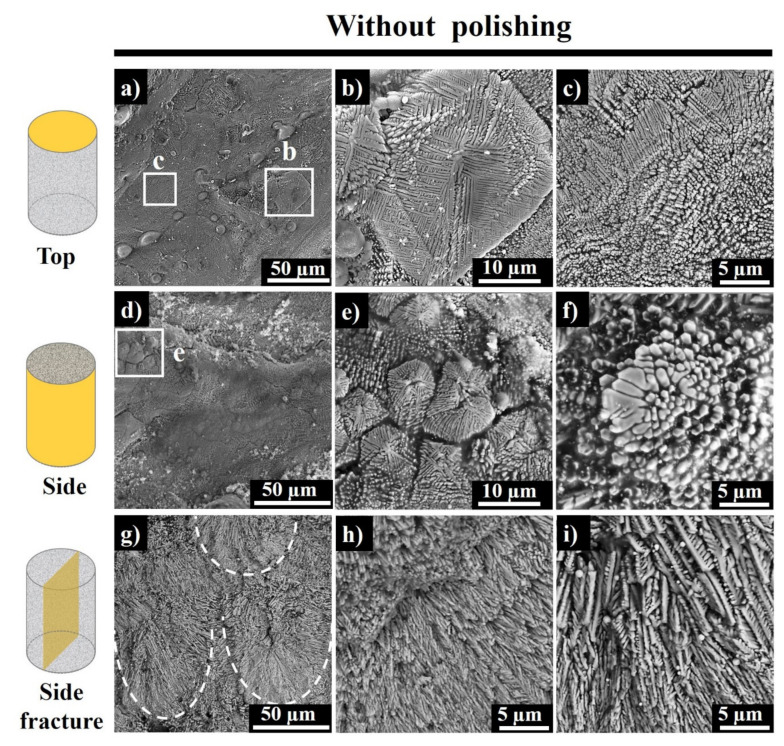
Top (**a**–**c**) and side (**d**–**f**) surface SEM images of as-built sample E9 and SEM images of unpolished side fracture (**g**–**i**) of E9 (white dashed ovals show the melt pools).

The SEM images of top and side surfaces of as-built sample E9 (150 W, 400 mm·s^−1^) and the unpolished side fracture are depicted in Figure 7. The distance between the center of the 2 scan tracks is approximately 90 µm, which corresponds to the set hatching space (85 µm). 

There are a few particles trapped in step edges, which represent the surface oxidized Al alloy particles (Figure 7a). Aluminum is susceptive to the formation of an alumina layer on the surface, which suppresses the melting procedure and subsequent wettability. 

Each scanned region of sample E9 consists of fine submicron particles and columnar dendritic structures. These fine particles are the tips of columnar dendrites grown parallel to build direction, while the long dendritic structures are formed perpendicular to the build direction (Figure 7c). Thus, there is a change in dendritic growth direction in the top printed layer. Large equiaxed dendrites with around 20 µm were found on the samples’ top surface (Figure 7b). Quite a few of the dendrites have secondary arms of 2–3 µm in size. The equiaxed dendritic crystals are originated from the undercooled molten mass when the latent heat of fusion is diffused across a cooler liquid forward to the intersection. Apparently, the temperature gradient is negative at the molten mass’ interface, whereas in the solidified area, it is about zero [32]. 

Figure 7d–f shows the side surface morphology of the as-built sample E9 along with the build direction. Each printed layer is composed of fine particles and dendritic structures, which is similar to the observed ones on the top surface as well. “Star-like” equiaxed dendritic structures with 5–10 µm dimensions were found on each scanned layer along with fine particles, which might represent the tips of the long dendrites grown perpendicular to build direction. 

Figure 7g–i shows the unpolished side fracture of sample E9. The area marked with white dashes illustrates the core of the melt pool with fine, columnar branched dendrites, while the surrounding overlapped regions of adjacent and consecutive melt pools have the comparatively coarser columnar dendrites due to the melt pool overlapping.

**Figure 8:**
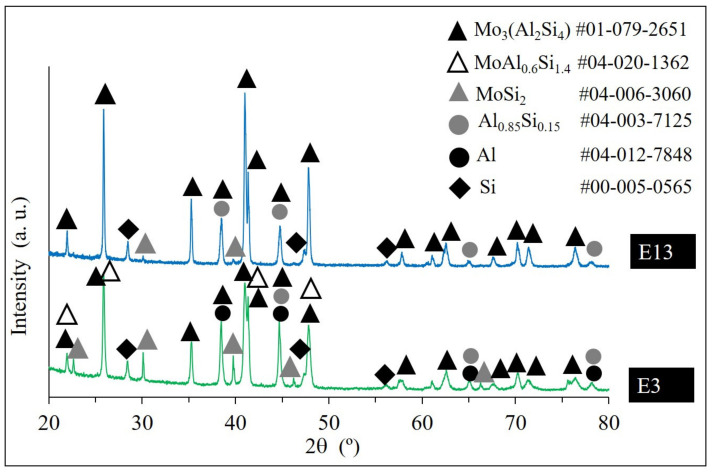
X-ray diffraction (XRD) patterns of samples E13 and E3.

Figure 8 shows the respective diffractograms of samples E13 and E3, when laser scan speed was fixed, but laser power was changed from 100 W to 250 W.

The X-ray diffraction (XRD) pattern of E13 indicates the characteristic peaks of Mo_3_(Al_2_Si_4_) phase (corresponding to Mo(Si_1−x_,Al_x_)_2_ composition, *x* = 0.33) with substituted Si and formed Al_0.85_Si_0.15_ phase, and weak peaks of unreacted MoSi_2_. In contrast to E13, the XRD pattern of E3 (laser power 100 W) shows that with a decrease in a laser power, the intensity of the unreacted MoSi_2_ peaks increases. Moreover, the XRD analysis revealed the coexisting hexagonal C40 Mo_3_(Al_2_Si_4_) and MoAl_0.6_Si_1.4_ (corresponding to Al unsaturated Mo(Si_1−x_,Al_x_)_2_, x = 0.3) phases. Conclusively, in case of a higher laser power, the completion of a single displacement reaction of MoSi_2_ and Al occurs and Al substitutes Si up to *x* = 0.33 mol. 

The effect of the scanning speed increase exhibits a similar trend. For sample E12, some amount of unreacted MoSi_2_ was detected, while in sample E9 remaining MoSi_2_ was not observed. Accordingly, the high scan speeds combined with the low laser powers result in chemical inhomogeneity.

**Figure 9:**
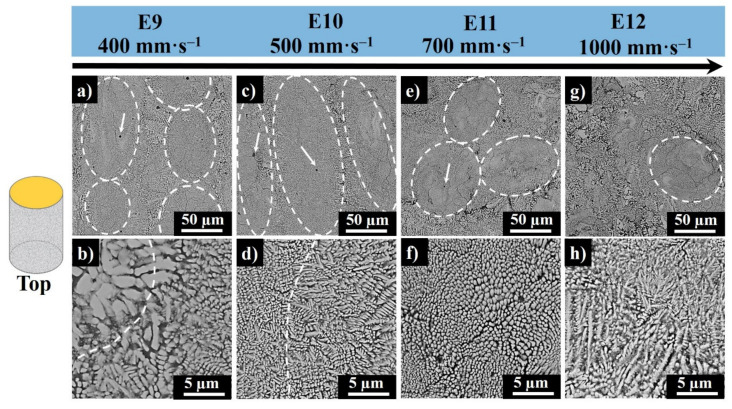
Top surface SEM images of E9 (**a**,**b**), E10 (**c**,**d**), E11 (**e**,**f**), and E12 (**g**,**h**) produced at 200W laser powers and at 400 mm·s^−1^, 500 mm·s^−1^, 700 mm·s^−1^, and 1000 mm·s^−1^ scanning speed, respectively (white dashed ovals represent melt pool cores, the white arrow points the precipitates of Al–Si rich phase, white dashes in b and d highlight the morphology transition zones).

Figure 9 shows the top surface SEM images of samples E9–E12. The top view micrographs demonstrate long scan lines composed of elliptical cross sections of the melt pool cores from the sequence of printed layers (marked with white dashed ovals). The change in microstructure highlights the melt pools in Figure 9. For samples E9 and E10, the melt pool core solidification mode is observed to be mostly columnar dendritic together with periodically cellular dendrites. The solidification mode for E11 is cellular and cellular dendritic. For E12, differentiation of the melt pools is rather difficult. This is because the process was out of the melting zone and the powder was sintered. Both E11 and E12 demonstrate the heterogeneity in microstructure caused by a fast scanning and not complete chemical reaction between MoSi_2_ and aluminum.

The melt pool cores in E9 represent the fine cellular or columnar dendrites of Mo_3_(Si_4_Al_2_) neighbored by a continuous network of hypoeutectic Al_0.85_Si_0.15_ in interdendritic regions, and Al has replaced the Si phase. In E10, the dendritic features are finer and, in E11, even smaller, conditioned by an increase in the scanning speed and a high cooling rate. The solidification mode of overlapping regions is columnar dendritic; thus, the core structure is enveloped with the coarser columnar dendritic crystals in the periphery.

The moving laser beam causes thermal gradients and growth rate variation in the center and the border of the melt pool, therefore the fineness of the Mo_3_(Al_2_Si_4_) grains in the core and the coarser grains at the border of the scan track as a higher cooling and solidification rates are associated with the core (see, e.g., Figure 9b,d). The columnar dendrites in E9 are misoriented, while in E10, they are well-oriented. The formation of columnar dendrites from cellular dendrites is affected by the thermal noise increase at the dendritic tips [33]. 

In all samples, fine and spherical precipitates of Al-Si rich phase of few micron size were observed (Figure 9, E9–E12, marked with white arrows). Moreover, form the top surface images, center-segregation of Al-Si rich phase was detected in the core of melt pools. 

**Figure 10:**
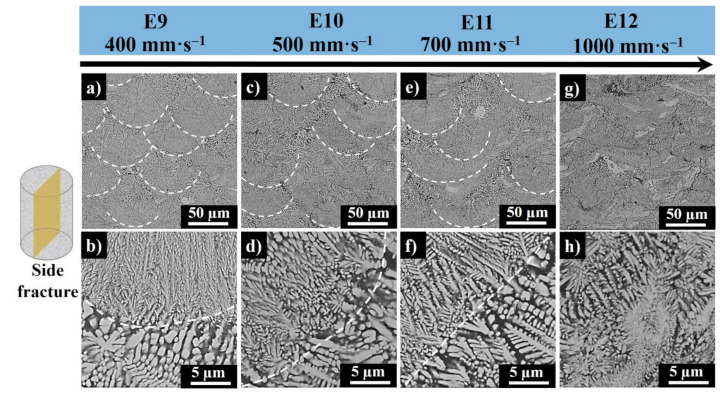
Side surface SEM images E9 (**a**,**b**), E10 (**c**,**d**), E11 (**e**,**f**), and E12 (**g**,**h**) produced at 200W laser powers and at 400 mm·s^−1^, 500 mm·s^−1^, 700 mm·s^−1^, and 1000 mm·s^−1^ scanning speed, respectively (the white dashed semi-ovals in panels (**a**–**g**) represent melt pools, and the white dashes in panels (**b**–**f**) show the morphology transition zone from melt pool core to edges).

Figure 10 represents the side surfaces of E9–E12. The semi-elliptical white lines depict the melt pool centers along to “Z” direction and perpendicular to the scan direction. The white dashes in Figure 10b,d,f,g indicate the morphology transition zone. The side fracture images of E9 (produced at the highest LVED) (Figure 10, E9) show that the vertical cross sections of the melt pool cores consist of well-oriented ultrafine columnar dendrites with secondary branches and casual ternary arming is grown parallel to the build direction following the maximum thermal gradient along the build direction [34]. In E10, the melt pools become slightly broader in the X–Y direction and narrower in the X–Z direction. In E11, a similar trend is noticed, and in E12, the melt pools were difficult to differentiate. Therefore, it can be concluded that the increase in laser scanning speed at a fixed laser power leads to a decrease in melt pool depth, which weakens the bonding between the consecutive layers, causing lack of fusion defects. The cores of the melt pools in samples E9–E11 are ringed by coarser columnar dendritic crystals in the overlapped areas of melt pool borders. The light gray regions found in sample E12 represent unreacted MoSi_2_ phase, and the dark gray regions represent Al- and Si-saturated phase.

**Figure 11:**
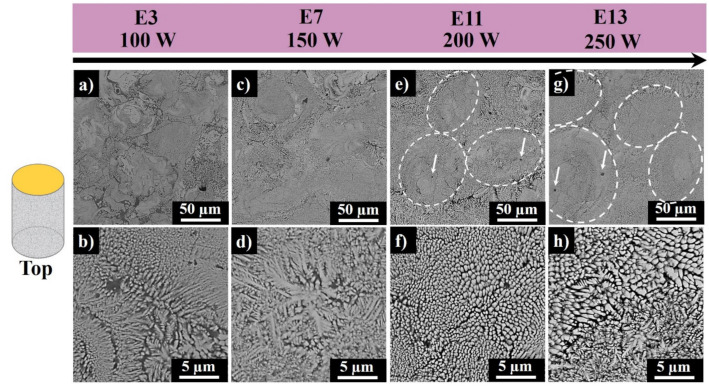
Top surface SEM images of sample E3 (**a**,**b**), E7 (**c**,**d**), E11 (**e**,**f**), and E13 (**g**,**h**) produced at 100 W, 150 W, 200 W, and 250 W laser powers, respectively, and at 700 mm·s^−1^ scanning speed (dashed ovals represent melt pool cores, and the white arrow points to the precipitates of Al–Si-rich phase).

Figure 11 shows the top surface SEM images of samples E3–E13, where laser scanning speed was kept at the fixed value of 700 mm·s^−1^, and the laser power was increased from 100 W to 250 W with 50 W step size. In E3 and E7 (produced at 100 W and 150 W laser power), different grain structures were observed ranging from cellular to equiaxed dendritic. The equiaxed dendrites mostly grow on the periphery of the melt pools due to incomplete melting of the previously spread powder layer. Fine round-shaped precipitates, highlighted by white arrows, were observed in sample E13, as well. As seen from Figure 11a–g, the microstructural homogeneity is improved with the increase in the laser power (from 100 W to 250 W).

The surface morphology of E13 represents the bimodal cellular and columnar dendritic structures. The columnar dendrites were nucleated and grown from the cellular grains due to the overlying of diffusive boundaries of cells leading to the dominance of columnar dendritic growth over the cellular ones.

**Figure 12:**
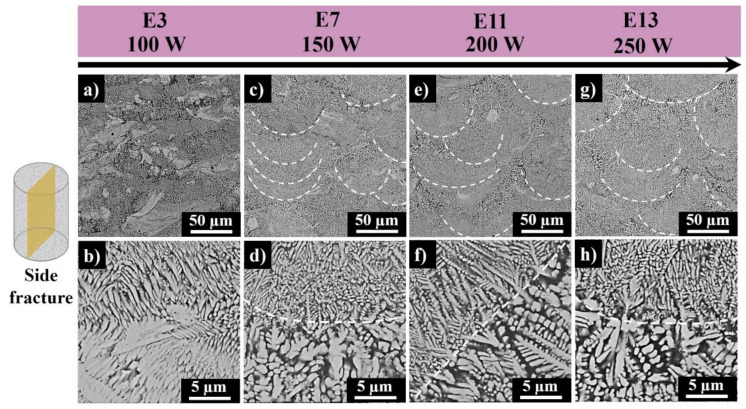
Side surface SEM images of sample E3 (**a**,**b**), E7 (**c**,**d**), E11 (**e**,**f**), and E13 (**g**,**h**) produced at 100 W, 150 W, 200 W, and 250 W laser powers, respectively, and at 700 mm·s^−1^ scanning speed (white dashed semi-ovals in panels (**c**–**g**) represent melt pools, and the white dashes in panels (**d**–**h**) show the morphology transition zone from melt pool core to edges).

Figure 12 shows the side fractures of samples E3–E13. For E3, the process went in a sintering mode as the apparent melt pool regions are not recognizable. For E7, the process went in a sintering/melting mode as the non-continuous melt pools are clearly visible. E11 and E13 have a more homogenous morphological texture as compared to sample E3 and E7. The finer regions (seen above a white dash line in Figure 12d–h) consist of fine columnar dendrites epitaxially grown parallel to the powder deposition direction. The region below the white dash is composed of the slightly disoriented coarser columnar dendrites. 

Conclusively, samples E9 and E10 prepared at 200 W laser power and 400–500 mm·s^−1^ scanning speed and sample E13 prepared at 250 W laser power and 700 mm·s^−1^ scan speed, show homogenous top microstructure and clearly expressed melt pools in the side. Accordingly, these parameters were considered as optimal to achieve the microstructurally and compositionally homogeneous materials. 

### 3.4. Density and Hardness

**Figure 13:**
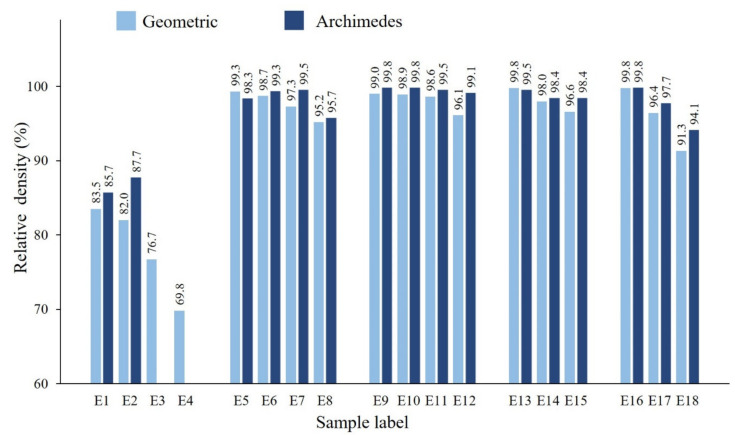
Relative geometric and Archimedes density results of E1–E18 samples (standard deviation is in the 0.1–0.4% range).

Figure 13 displays the geometric and Archimedes density values and the corresponding relative density results. For E3 and E4, the Archimedes density was not evaluated because of the huge amount of open porosity. An increase in the scan speed in all five sets results in a gradual decrease in density, as the applied volumetric energy decreases with an increase in the scan speed (Figure 13). 

For the samples in columns I–V, Figure 1, where the laser power is increased with a step size of 50 W, the density is gradually increased, as the applied energy promoted the complete melting of the deposited layers and the sufficient bonding between consecutive layers. 

Samples E15, E17, and E18 produced using the high scanning speeds (1250–1500 mm·s^−1^), have a relatively low density being almost insensitive to the laser power, nonetheless greatly dependent on the scan speed. Moreover, though samples E2, E12, E15, and E18 were produced using the same LED (67.2 J·mm^−3^), but sample E2 demonstrates the least densification followed by sample E18. 

By optimizing process parameters samples possessing up to 99.8% relative density were manufactured. 

It is well documented that the hardness depends not only on density, but also on developed phases; solidification mode; and respective microstructure of phase constituents, grain size, morphology, and phase segregation [35,36]. To ensure the reliability of hardness results, both HV1 and HV5 were taken into consideration.

**Figure 14:**
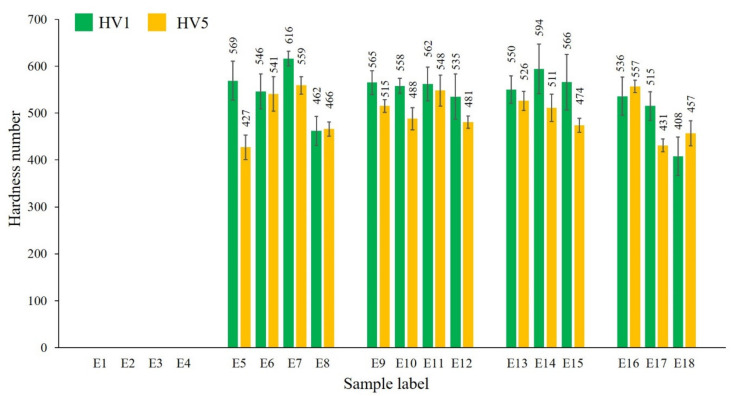
Hardness results of samples E1–E18, averaged for 10 indentations.

Because of high porosity, the hardness of samples E1–E4 (Figure 14) was not measured. Unreacted MoSi_2_ remained during scanning is responsible for insignificant fluctuations in hardness measured for materials produced at the same laser power. 

The standard deviation bars show the higher fluctuations in the HV1 results, because of the small size of indentation marks. The heterogeneous microstructure of E8 and E18 is responsible for a relatively low hardness of 500 ± 57 HV1. All other samples exhibit hardness of up to ~600 HV1 and ~560 HV5. 

## 4. Conclusions

The composites of Mo(Si_1−x_,Al_x_)_2_, x = 0.3–0.33 mol, were successfully manufactured by reactive LPBF of fairly flowable MoSi_2_-30 wt.% AlSi10Mg powder mixture.. The study of the process parameters in terms of laser power and laser scanning speed was performed to optimize the LPBF processing of mechanically reliable and microstructurally homogeneous materials for possible industrial applications where materials of high resistance to the pest oxidation are needed. The applied conditions were changed in the wide range of the laser power and the scanning speed of 100–300 W and 400–1500 mm·s^−1^, respectively. It was shown that the samples produced at 150–200 W laser power and 400–500 mm·s^−1^ scanning speed, as well as at 250 W laser power and 700 mm·s^−1^ scanning speed, possess an admissible surface finish and the notable chemical and microstructural homogeneity. The longitudinal cross section analysis of the melt pools discloses the fine columnar dendritic structures of Mo(Si_1−x_,Al_x_)_2_ phase encircled with the coarser dendrites along with interdendritic hypoeutectic Al_0.85_Si_0.15_ phase and substituted Si. It was revealed that an increase in the laser scanning speed at a fixed laser power and a decrease in the laser power at a fixed scan speed result in the density reduction, as well as in the chemical and microstructural inhomogeneity. 

The recommended LPBF process parameters (for the LPBF setup indicated in the experimental section of this paper) to produce the industrially applicable materials can be specified as the following.

- Laser power in the range of 150 to 250 W
- Scanning speed in the range of 400 to 700 mm·s^−1^
- Layer thickness: 35 µm
- Hatching distance: 85 µm
